# Ecological insights on the feeding behaviour of waterbirds in an Important Bird and Biodiversity Area of South West Johor Coast, Malaysia

**DOI:** 10.3897/BDJ.13.e141250

**Published:** 2025-01-15

**Authors:** Nur Athirah Fauzi, Kaviarasu Munian, Nur Aina Amira Mahyudin, Nor Atiqah Norazlimi

**Affiliations:** 1 Zoology Branch, Forest Biodiversity Division, Forest Research Institute Malaysia (FRIM), 52109, Kepong, Selangor, Malaysia Zoology Branch, Forest Biodiversity Division, Forest Research Institute Malaysia (FRIM) 52109, Kepong, Selangor Malaysia; 2 Environmental Management and Conservation Research Unit (eNCORe), Faculty of Applied Sciences and Technology, Universiti Tun Hussein Onn Malaysia (Pagoh Campus), Johor, Malaysia Environmental Management and Conservation Research Unit (eNCORe), Faculty of Applied Sciences and Technology, Universiti Tun Hussein Onn Malaysia (Pagoh Campus) Johor Malaysia; 3 Faculty of Applied Sciences and Technology, Universiti Tun Hussein Onn (Pagoh Campus), KM 1, Jalan Panchor, 84600 Pagoh, Johor, Malaysia Faculty of Applied Sciences and Technology, Universiti Tun Hussein Onn (Pagoh Campus), KM 1, Jalan Panchor, 84600 Pagoh Johor Malaysia

**Keywords:** Important Bird and Biodiversity Area (IBA), feeding behaviour, intertidal, Scolopacidae, waterbirds

## Abstract

Mangroves and mudflats are essential intertidal habitats that support benthic communities, providing critical feeding grounds for waterbirds. However, the degradation of these habitats due to coastal reclamation poses significant threats to prey availability and waterbird populations along the South est Johor Coast. While most avian research in Johor focuses on forest birds, studies on coastal waterbirds, particularly their feeding ecology, remain scarce. Understanding the feeding ecology of waterbirds is crucial for strengthening conservation efforts in vulnerable intertidal habitats. This study investigated the feeding behaviour and diet composition of waterbirds along the South West Johor Coast, Malaysia. Fieldwork was conducted in three coastal mudflats from November 2020 to May 2021, employing the direct observation technique. A total of 576 hours of observation were recorded, averaging 2 hours and 19 minutes per focal observation. Of 17 waterbird species recorded, only 11 species were included in the analysis based on sufficient data. The results revealed that waterbirds primarily consumed fish, molluscs, worms, crabs and unidentified prey, with fish comprising 25% of their overall diet. Feeding behaviour varied significantly by morphology traits, with larger waterbirds demonstrating higher feeding efficiency. Despite lower feeding rates and shorter feeding durations, larger species had a greater percentage of successful feeding attempts, indicating their superior ability to meet energy requirements. These findings provide crucial baseline data for understanding waterbird feeding ecology and highlight the importance of conserving the intertidal habitats. This research contributes to the development of targeted conservation strategies for waterbirds in the Important Bird and Biodiversity Area (IBA) of the South West Johor Coast, Malaysia, an area increasingly at risk from habitat degradation.

## Introduction

Intertidal mudflats have been valued for their ecosystem functions and services, serving as a foundation for conservation initiatives over the past decades (Cabello et al. 2012). These habitats are critical for maintaining biodiversity and supporting numerous ecosystem services that benefit both wildlife and humans. For instance, mudflats act as feeding grounds for migratory birds, particularly waterbirds, which rely on the rich benthic communities for sustenance during long migrations (Delany et al. 2009). These mudflats are also highly productive habitats for bottom-dwelling invertebrates, such as mussels, horseshoe crabs and mud snails and, thus, provide food for predatory fish and birds (Christianen et al. 2017). The diverse ecological functions provided by intertidal wetlands have unfortunately led to their extensive exploitation (United Nations Environment Programme (UNEP) 2001). According to a report by the International Union for Conservation of Nature (IUCN), the destruction of intertidal zones in East and Southeast Asia is contributing to some of the most rapid biodiversity losses globally (MacKinnon et al. 2012). This loss is particularly evident in the significant decline of globally-threatened bird species, especially waterbirds (Birdlife International 2001). Current estimates suggest that the extent of intertidal habitat loss in Asia is comparable to or exceeds the global decline of mangroves and coral reefs (Millennium Ecosystem Assessment 2005).

Waterbirds are essential components of wetland ecosystems, utilising various habitats for nesting, breeding, foraging, stopover and wintering during migration (Saygili et al. 2011). These species are typically found in intertidal or inland wetlands, where they have evolved physical and behavioural adaptations for life in or near water. Unlike other birds, waterbirds forage by wading in shallow waters, probing mud or sand with their bills. Their long legs allow them to navigate shallow areas while keeping their bodies dry, facilitating feeding, while their bills vary in shape and size, each suited to a specific feeding strategy (Olsen 2017). As migratory species, waterbirds rely heavily on staging sites to replenish energy reserves during long migrations (Hua et al. 2013). Habitat degradation along migratory corridors is directly linked to the decline in waterbird populations (Studds et al. 2017). Intertidal zones, amongst the most threatened habitats globally, are rapidly disappearing, with nearly half of these habitats lost due to human expansion (Silva et al. 2007). Van de Kam et al. (2004) noted that many waterbird species are highly site-dependent, unable to adapt or relocate their feeding and nesting areas. Those that do relocate face increased competition, leading to higher mortality and reduced fitness. Over time, waterbirds may become opportunistic, exploiting suboptimal habitats such as abandoned mines, lakes and ponds as available foraging and nesting sites along intertidal zones decline (Ismail and Rahman 2012).

In Malaysia, intertidal flats cover an estimated 52,951 hectares or roughly 1% of the nation’s total wetland area, with the majority located in the states of Selangor and Johor (Parlan et al. 2021). However, the destruction of coastal habitats has been significant, with the rate of loss estimated to have increased by approximately 1% or 1,282 hectares per year, since 1990 (Omar 2012). Major drivers of coastal land-use change include industrialisation, urbanisation, agricultural expansion and human settlement (Clark 2018). The East-Asian Australasian Flyway (EAAF), passing through Malaysia, is the most threatened migratory route, with the highest number of globally-threatened species (Boere et al. 2006). Migratory waterbird populations along the EAAF have decreased by 48%, with an annual decline up to 8% (International Wader Study Group 2003, Amano et al. 2010, Studds et al. 2017). The decline of wetlands has become a prominent concern for conservationists, scientists, policy-makers and the public. In response, government agencies, non-governmental organisations and international bodies have undertaken various initiatives to conserve waterbirds and their habitats. In Malaysia, wetlands are protected under multiple legal policies and laws, with the level of protection and the responsible agency varying depending on the specific legislation applied.

Apart from policies and laws, the national spatial planning of development incorporated coastal wetlands as one of the environmentally sensitive areas (ESAs) under the National Physical Plan (NPP). The NPP defines ESAs based on environmental, physical, cultural and climatic criteria and mandates that these areas remain undisturbed. Preservation or sustainable management of ESAs is required, guided by their type, sensitivity and ecological significance (PLANMalaysia 2017). However, despite these protective measures, significant gaps remain in the understanding of ecological dynamics, particularly for waterbirds in coastal regions.

In this context, research on waterbirds in Malaysia has predominantly focused on non-coastal wetlands (Ismail et al. 2012, Ismail and Rahman 2012, Rajpar and Zakaria 2012, Rajpar and Zakaria 2013, Rajpar and Zakaria 2014). Coastal studies have mainly provided basic data on species diversity and distribution through inventories and censuses. Although the Malaysian Nature Society (MNS) has compiled extensive data on waterbird distribution and abundance along the South West Johor Coast, many ecological aspects remain underexplored. The majority of data assessments in Malaysia concentrated on species or family diversity, often neglecting critical factors, such as the distribution of waterbirds and their relationship with environmental variables. Furthermore, comprehensive studies on the feeding ecology of coastal waterbirds remain scarce. Only a few recent investigations have examined this aspect, such as studies by Ismail and Rahman (2016) in the Kuala Gula Bird Sanctuary and Norazlimi and Ramli (2015) in Pantai Jeram and Pantai Remis, Selangor. Although research has been conducted on bird composition and diversity in Tanjung Piai National Park (Yatim-Mustafar et al. 2019, Fauzi et al. 2023) and coastal birds in Tanjung Laboh (Mokhter et al. 2021, Jien et al. 2021, Fauzi and Norazlimi 2021), these studies have not addressed the feeding ecology of waterbirds.

Notably, there is a significant gap in knowledge regarding the diets, foraging rates and prey availability for waterbirds in this region. Understanding the feeding ecology is intrinsically linked to population dynamics and provides insights into trophic interactions, such as prey selection, evolutionary adaptations, predation and energy transfer within and across ecosystems (Braga et al. 2012). Feeding ecology encompasses key elements such as food selection, habitat preferences and prey-capturing techniques or types of behaviour specific to avian species in a given habitat (Aboushiba et al. 2013). Accordingly, this study aims to examine the feeding behaviour and dietary composition of waterbird populations inhabiting three mudflats along the South West Johor Coast.

## Materials and methods

### Study area

**Figure 1.**
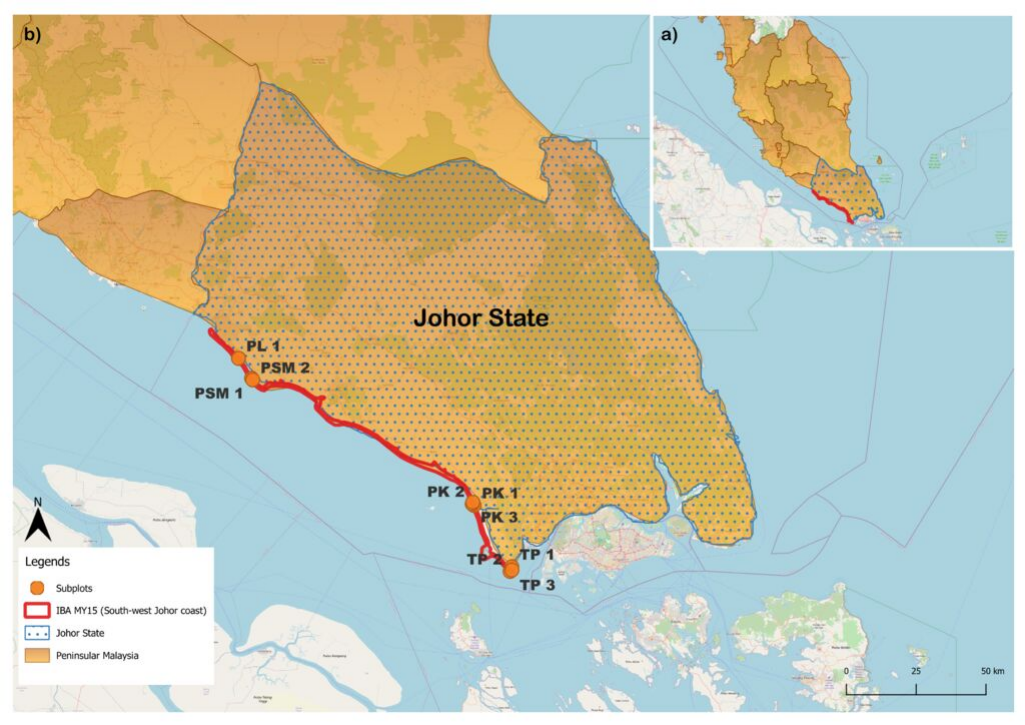
Location of study sites along the Important Bird and Biodiversity Area of South West Johor Coast.

The study was focused on the mudflat areas along the IBA in the southern part of Peninsular Malaysia, namely South West Johor Coast. Three study sites have been chosen within two districts (Pontian and Muar), which are (i) Tanjung Piai National Park, (ii) Pontian Kechil coastal areas, and (iii) Pantai Leka and Pantai Mesra Seri Menanti, Muar (Fig. 1) (Yeap et al. 2007). The coordinates and protection status of each study site were summarised in Table 1. The study sites were chosen according to the highly reported waterbird presence based on previous studies and the annual report of the Asian Waterbird Census and were shown to have received different intensities of coastal reclamation activities. From north to south, the point distance between Muar and Pontian Kechil is approximately 98.87 km, while, from Pontian Kechil to Tanjung Piai, it is approximately 27.02 km.

**Figure 2.**
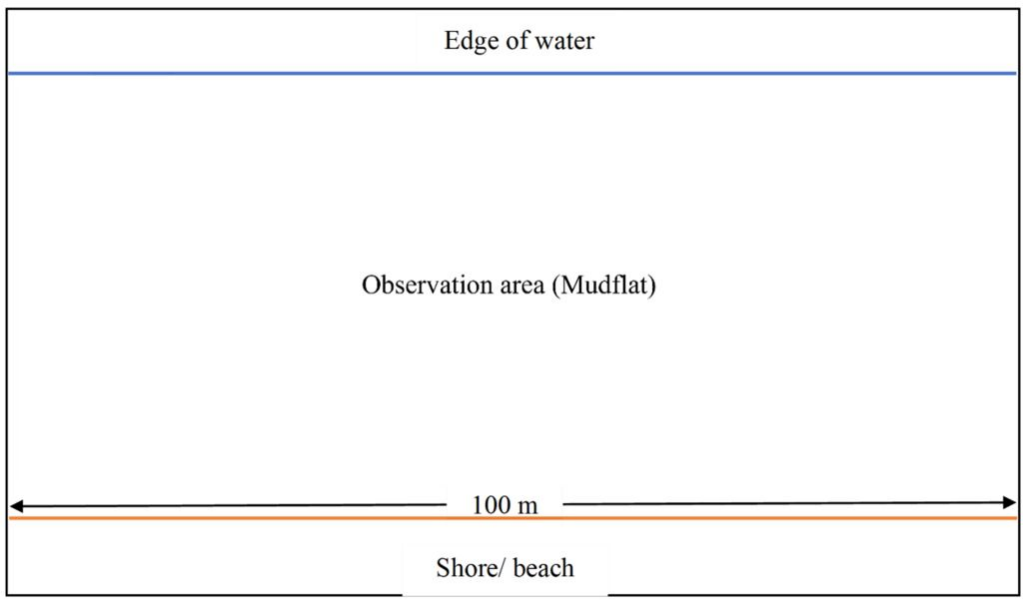
General design of the observation plots in all study areas.

A standardised sampling protocol for each site was carried out with the same equipment and tools with the same sampling duration. A total of nine study plots, situated specifically on tidal flats of the three study sites, with three plots for each study site, were chosen for waterbird observations. The size of each observation plot is approximately 100 m in length and the width is according to the distance between the shore and the edge of water that ranged from 0 m to 700 m, as tidal flats are always affected by tidal cycles (Colwell and Sundeen 2000), (Fig. 2).

### Feeding behaviour observation

The observations were commenced from November 2020 until May 2021. At each site, three plots were established and two observation sessions were carried out for six days period. Surveys were alternated amongst the three plots within each site to ensure comprehensive spatial coverage. The observations were done during the Movement Control Order phase during the COVID-19 outbreak, which restricted the number of observers for the study to two personnel.

To minimise observer bias, the same two observers participated in all sessions, underwent standardised training in bird identification and data recording and resolved any discrepancies collaboratively, based on established field guides and protocols. The study focused on migratory phases, the southwards migration from June to December and the northwards migration occurring from February to May. Observations were conducted in four time intervals: 0800 to 1000 hours, 1000 to 1200 hours, 1400 to 1600 hours and 1600 to 1800 hours. The interval between 1200 and 1400 hours was excluded from the study due to the lack of waterbird activity, as determined through preliminary sampling. This systematic approach produced detailed insights into waterbird feeding behaviour across different migration periods.

The detailed feeding behaviour of waterbirds in three study sites was observed through focal observation with the aid of spotting scopes (Nikon 13-30 x 50 mm ED Angled). In addition, some of the observations were recorded using a DSLR Camera (Nikon D5500) with a Nikkor lens (Nikon 200-500 mm F/5.6E ED VR AF-S) when possible. A stopwatch was used for each focal observation to determine the time spent by waterbirds during feeding. The observation was conducted during the low tide period only. This reduces the bias in observation, as only large waterbirds can forage during high tide since they have longer legs. In each focal observation, an individual was selected from a flock. The selected waterbird needed to be actively feeding from the time the waterbird began actively searching for prey until the prey was completely swallowed. If the selected waterbird left the observation area in the span of 30 seconds, the observation was discarded from the analysis (Burger et al. 2007).

To avoid multiple observations of the same individual, the next selected waterbird needed to be located at least 10 m away from the previously observed individual. However, when there were more than one species of waterbird feeding simultaneously within the same observation area, the next waterbird for observation needed to be selected from a different species flock. The behavioural record was based on the method reported by Norazlimi and Ramli (2015). In each focal observation, the following data were collected:

a) Pecks per minute: Pecks are defined as the feeding attempts when a waterbird is collecting prey from the surface of the substrate. These data were measured in this study to calculate the feeding rate and percentage of successful attempts by waterbird species. The feeding rate is the total number of feeding attempts (pecks or probes) made by waterbirds per minute. Meanwhile, the percentage of successful attempts was calculated by dividing the number of successful attempts that consumed the prey per minute by the feeding rate per minute and then multiplying the value by 100 to get the percentage. Prey items per minute were then used to determine the success rate. The success rate is the number of prey items consumed by each observed waterbird per minute. The summarised formula is as follows:

\begin{varwidth}{50in}\begin{equation*}
            \text{Percentage of successful attempts (\%)} = \left (\frac{\text{Number of successful attempts (per minute)}}{\text{Feeding rate (per minute)}} \right) \times 100
        \end{equation*}\end{varwidth}

b) Prey types consumed: The types of prey were classified into fish, mollusc, worm, crab and unknown. Prey that cannot be seen clearly during observation were classified into the unknown group. This group of data was used to determine the diet composition of each waterbird species.

c) Time spent foraging: The time was measured starting from when the waterbird was actively searching for its prey until the prey was swallowed entirely (estimates were recorded in minutes).

d) Flocking behaviour: The waterbirds were classified as solitary if they were observed foraging alone or separate from other waterbirds. In contrast, the waterbirds were classified as an intraspecific flock if they were foraging in a group or flock of the same species, while the waterbirds foraging in the mixed-species flock were classified as an interspecific flock.

e) Foraging techniques: The foraging techniques engaged by waterbirds were classified into three types:

1. Tactile hunting technique: individuals forage as they walk, with bills probing continuously into the substrate (Baker and Baker 1973, Gerritsen and Sevenster 1985);
2. Visual-feeding technique: individuals forage in a continuous manner and peck at the items seen on the substrate surface (Anderson 1981);
3. Pause-travel technique: individuals forage by scanning the area in front of them and pecking at the substrate surface when prey is detected in a stop-run-stop pattern (Pienkowski 1983, Metcalfe 1985).

### Data analysis

All the data collected were organised using the Microsoft Excel software. For the analysis, Paleontological Statistic (PAST) (Hammer and Harper 2006) and RStudio (RStudio Team 2020) were used. All the datasets were tested with the Kolmogorov-Smirnov test for normality as the analysis was comprised of a large sample size (n ≥ 50) (Mishra et al. 2019).

The average time spent feeding for each species was calculated by dividing the total time spent feeding by each species by the total number of individuals observed for each species. The differences in foraging behaviour between species and behavioural categories were compared using the Kruskal Wallis test because the data collected were not normal. The non-parametric multiple comparison test was used when the Kruskal-Wallis test indicated a significant difference.

In addition, the Spearman Correlation test was conducted to test the relationship between time spent feeding by the waterbirds and their success rate, followed by the relationship between feeding rate and success rate of waterbirds. The Spearman Correlation test measures the strength and direction of association between two variables. The test produce an R-value ranging from +1 to -1. R-value of +1 indicates a perfect positive association, while the value of 0 indicates no association and -1 indicates a perfect negative association. The closer the R-value is to 0, the weaker the association between the two variables.

## Results

**Figure 3.**
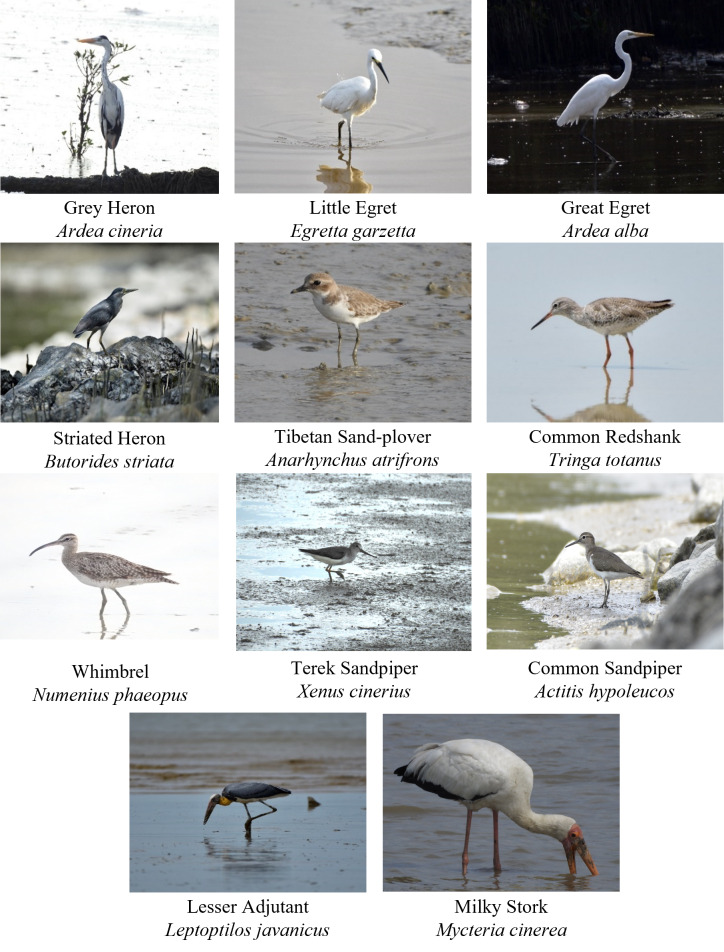
Waterbird species observed in study sites.

The total observation hours were 576 hours, with an average of 2 hours and 19 minutes spent for each focal observation for 11 species. According to a previous study by Lendvai et al. (2015), one hour of observation is sufficient to reflect the variation in feeding behaviour amongst individuals reliably. The study also suggested that one hour was, in fact, the optimal sampling time, given that it maximised accuracy while minimising total sampling effort. A total of 355 focal observations on the feeding behaviour of waterbirds were recorded across three study sites. The overall focal observations involved 17 waterbird species. However, only 11 species with sufficient total observation hours (more than one hour) were included in the analysis (Table 2), (Fig. 3). Amongst these 11 species included in the analysis, they were species from four families such as Ardeidae, Charadriidae, Scolopacidae and Ciconiidae.

### Diet composition of waterbirds

In terms of diet composition, five prey groups, namely fish, mollusc, worms, crabs and unknown groups, were observed as the main diet for waterbirds. Based on the observation, the fish group was the most preferred diet amongst all the waterbird species, accounting for a total of 25% of the waterbirds’ diet, followed by worms (23%), unknown (22%), mollusc (19%) and crab (11%). The prey items categorised under the unknown group were the prey that could not be seen and identified during the observations.

**Figure 4.**
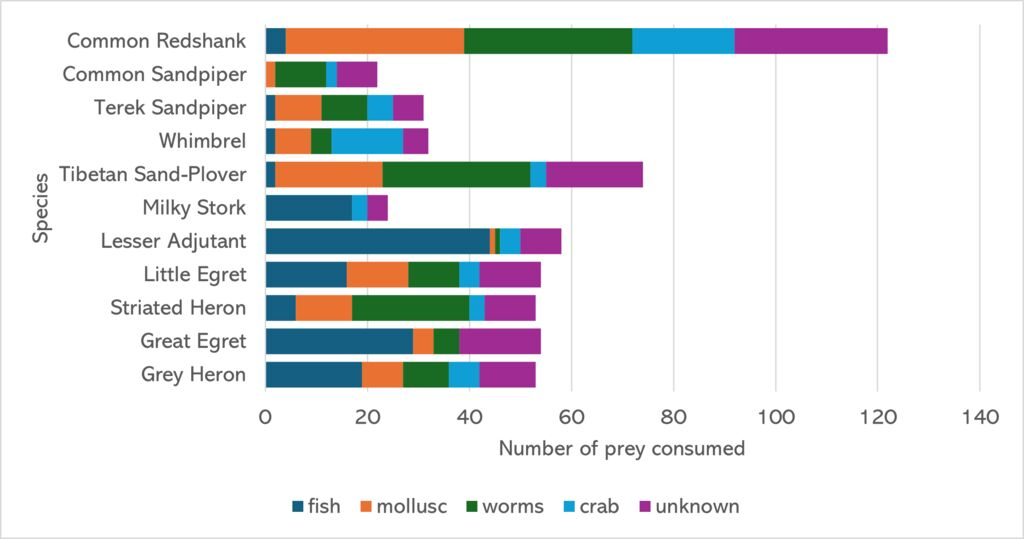
The diet composition of each waterbird species, based on 576 hours of observation.

All the observed species consumed fish, except for Common Sandpiper (*Actitishypoleucos*), Terek Sandpiper (*Xenuscinereus)* and Tibetan Sand-plover (*Anarhynchusatrifrons)*. Fish were also determined to be the main diet for large waterbirds, such as Milky Stork (*Mycteriacineria*), Lesser Adjutant (*Leptoptilosjavanicus*), Little Egret (*Egrettagarzetta*), Great Egret (*Ardeaalba)* and Grey Heron (*Ardeacinerea*). Meanwhile, worms were mainly consumed by Common Redshank (*Tringatotanus*), Common Sandpiper (*Actitishypoleucos*), Tibetan Sand-plover (*Anarhynchusatrifrons*) and Striated Heron (*Butoridesstriata*). Worms and mollusc were observed in the diet of all the waterbird species, except for Milky Stork, with Common Redshank consuming them most frequently (Fig. 4).

### Feeding techniques

In this study, waterbird species were observed to engage with different feeding techniques. However, there was a similar pattern in the preferred feeding techniques amongst the waterbirds with the same body size. Three feeding techniques were observed to be practised by waterbird species. All the individuals of the Striated Heron were practising the pause-travel technique, while the Whimbrel, Terek Sandpiper and Common Sandpiper were observed to practise the tactile-hunting technique solely. Grey Heron and Great Egret preferred to practise the pause-travel technique, although a small population of the individuals used the visual-feeding technique. In contrast, Little Egret, Lesser Adjutant, Common Redshank and Tibetan Sand-plover were seen to engage in both pause-travel and tactile hunting techniques. However, the pause-travel technique was the most preferred by Little Egret and Lesser Adjutant, while the Common Redshank and Tibetan Sand-plover opted more for the tactile-hunting technique. Only Milky Stork was observed to practise all three feeding techniques equally. There was a significant difference between the time spent on feeding and the feeding techniques engaged by the waterbirds (H = 5.665, p = 0.047). The pairwise comparison test showed that the differences lie between the visual-feeding technique and the pause-travel technique (p = 0.045), as well as between the visual-feeding and tactile-hunting techniques (p = 0.024) (Table 3).

### Time spent feeding

**Figure 5.**
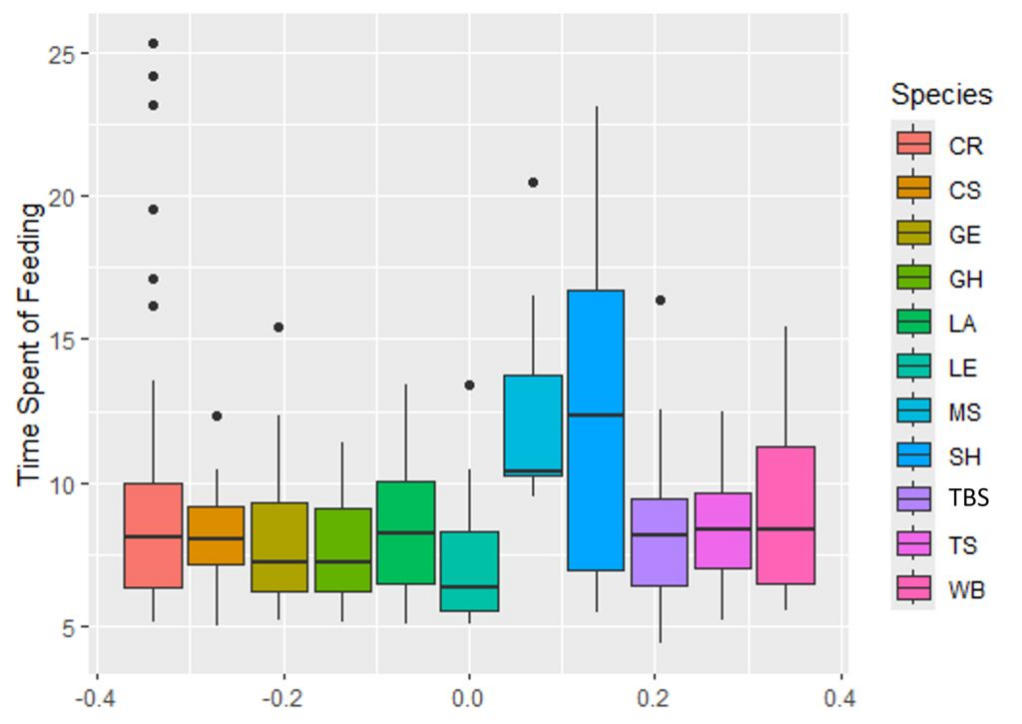
The average time spent feeding for each waterbird species. Common Redshank (CR), Tibetan Sand-plover (TBS), Little Egret (LE), Grey Heron (GH), Milky Stork (MS), Whimbrel (WB), Terek Sandpiper (TS), Common Sandpiper (CS).

The time spent feeding for each waterbird individual was recorded and the average minutes spent during feeding were calculated (Fig. 5). Milky Stork and Striated Heron were observed to be spending the longest time feeding, with average durations of 10.16 minutes and 12.26 minutes, respectively. Meanwhile, Little Egret spent the shortest feeding time, with an average duration of 7.19 minutes. As detected by the Kruskal-Wallis test, there was a significant difference of time spent feeding between the species (F = 7.22, p = 2.82E-10). Meanwhile, the Spearman correlation test showed no association between the feeding time spent by waterbirds and their success rate (R = 0.27, p = 0.418). However, based on the observation, the waterbirds such as Common Redshank, Common Sandpiper, Tibetan Sand-plover and Terek Sandpiper with the highest success rate usually spend about eight to ten minutes for feeding (Table 4).

### Flocking behaviour

**Figure 6.**
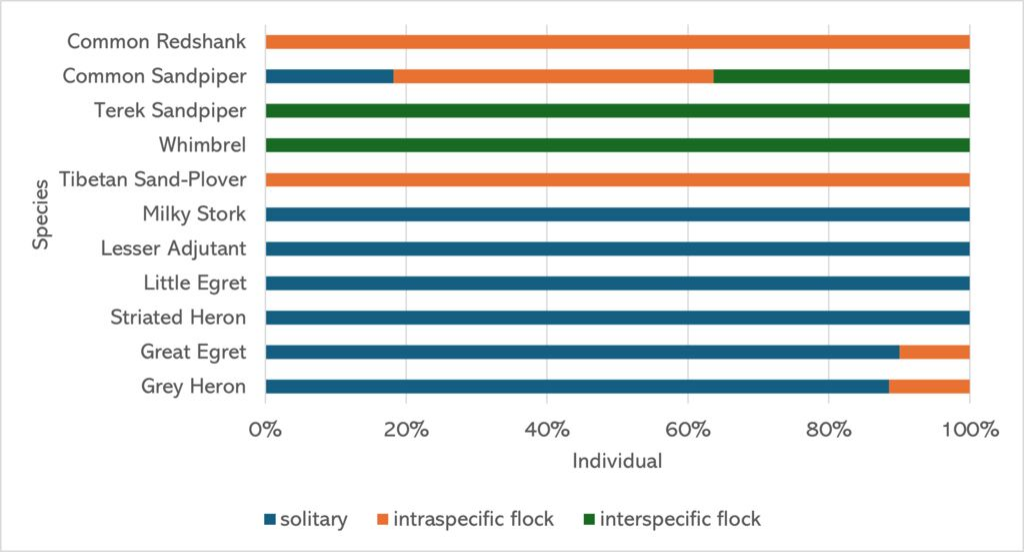
The percentage of waterbirds practising three different types of flocking behaviour.

Although most waterbird species exhibit a distinct flocking behaviour, observational data show that 90% of Great Egrets and 88% of Grey Herons were recorded feeding individually (solitarily). In contrast, the rest of the individuals were observed in a group of the same species, also known as intraspecific flocks (Fig. 6). Intraspecific flocks are also common in smaller waterbird species, such as Common Redshank and Tibetan Sand-plover. Whimbrel and Terek Sandpipers were also observed feeding near or mixed within these flocks (interspecific flocks). Meanwhile, larger waterbirds like Milky Stork, Lesser Adjutant, Little Egret and Striated Heron, were commonly known as solitary feeders. Only the Common Sandpiper species were observed to practise all three kinds of flocking behaviour. The interaction of waterbirds foraging within a flock showed more aggression and competition with each individual. These kinds of behaviour were observed in the flocks of Common Redshank and Tibetan Sand-plover, where they usually fought for the same prey resources.

### Feeding rate, success rate and percentage of successful attempts

Feeding success is crucial for waterbirds, mainly maintaining their body fitness and fuelling their energy supply before the long-distance migration and breeding activities (Battley et al. 2003, Battley and Piersma 2005, Finn 2010, Schmaljohann et al. 2022). This study revealed that the feeding rate, success rate and percentage of successful attempts were highly influenced by the differences between the waterbird’s morphological traits, feeding techniques engaged and flocking behaviour, factors that are closely related to their time spent feeding. The Kruskal-Wallis test showed a significant difference in feeding rate values obtained between species (H = 89.4, p = 0.002). Pairwise comparison supported the previous statement by proving the differences that occurred between species, for example, Striated Heron and Tibetan Sand-plover (z = 73.8, p = 0.002) and Striated Heron and Common Redshank (z = 89.4, p = 0.032).

Based on these observations, Common Redshank and Tibetan Sand-plover had the highest feeding rates compared to other species (Table 4). Meanwhile, Striated Heron recorded the lowest feeding rate compared to other species, as the average number of feeding attempts (by pecking or probing) per minute was lower for this species. As for this, Striated Heron was observed to practise the pause-travel technique, requiring extra time for searching and scanning its prey items before capturing it. Although Striated Herons only made an average of one feeding attempt per minute, most of their attempts successfully captured the prey, contributing to the highest percentage of successful attempts (90%) compared to other species. The pause-travel techniques engaged by the species are likely associated with the movement from one micro-patch to another (Dimalexis et al. 1997) that can prevent the prey from running away and ensure success in every attempt made by the species.

**Figure 7.**
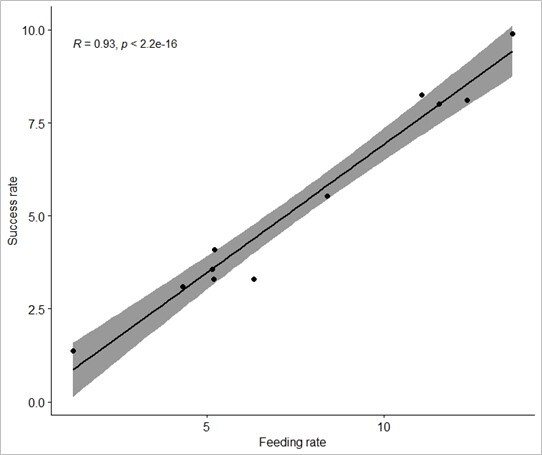
The positive correlation between the feeding rate and the success rate of observed waterbirds.

A significant difference was observed in success rate between species by Kruskal Wallis test (H = 2.87, p = 0.046). Common Redshank recorded the highest success rate and measured the highest feeding rate. Spearman correlation analysis was conducted to test the relationship between feeding and success rates (R = 0.927, p < 0.05). The R-value was close to 1, thus indicating a strong positive correlation since the feeding rate increases with the success rate (Fig. 7).

## Discussion

The findings of this study lies in combining both direct field observations of species-specific behaviors (e.g., Terek Sandpipers and Whimbrels handling large crabs, Striated Herons’ pause-travel tactics) with insights into how morphological differences influence feeding success. By highlighting these connections, our work addresses a critical gap in previous research, where emphasis often focused on abundance and distribution without fully exploring how anatomical and behavioral adaptations determine foraging efficiency. Understanding these nuances is essential for interpreting how different species meet their energetic needs within intertidal environments and can inform broader conservation strategies aimed at maintaining functional diversity in waterbird populations. Moreover, these individual foraging adaptations intersect with social behaviors, such as flocking, which can further modify feeding success and predator avoidance.

Feeding behaviour and prey selection by waterbirds are heavily influenced by their morphology, particularly the morphology of bill length and shape (Zeffer et al. 2003, Nebel et al. 2005, Mathot et al. 2018). Based on the observations, small waterbird species, such as Tibetan Sand-plover, Common Sandpiper and Common Redshank, were found to consume smaller prey items as compared to other large species. With their small and short bill, their diets are mainly comprised of small prey, including molluscs and worms. Shorter-billed waterbirds tend to insert fully or most of their bills into the mud while pecking for prey and primarily take surface-living or shallow-dwelling invertebrates (Piersma and Wiersma 1996, Piersma et al. 1996). These species were also seen pecking on small crabs crawling on the mud surface that suits their bill size. Small crabs are usually extremely abundant on the surface of the mudflats during low tides, while larger crabs only allow themselves to go out from the burrow for food.

On the other hand, although Whimbrel and Terek Sandpiper can be considered small-bodied waterbirds, both species have a long, unique-shaped bill that curves upward or downward. The special bill structure allows them to catch larger prey than small-bodied waterbirds with shorter bills. Large crabs are a major prey item in the diet of Whimbrel and Terek Sandpiper species observed in the overall study sites. Ferns and Siman (1994) reported that the curved bill of waterbirds provides a significant advantage in probing the crabs that usually escape and hide in their burrow. A curved bill is more readily manipulated than a straight bill along the complex capture path, which follows the burrow system within the sediments (Ferns and Siman 1994, Van de Kam et al. 2004). The species with the curved bill can change their direction by rotating their head or neck rather than moving their entire neck and head only. The only drawback of the curved bill is that it is not as structurally strong as a straight bill (Burton 1974, Owen 1984). As a result of the alteration of bone and muscle to strengthen the curved bill, there is a reduction in the relative length of the tongue, which averts the transport of prey along the bill while it is still inserted in the sediment (Burton 1986). Hence, the prey must be removed from the sediment before the waterbird can swallow it.

There are debates that still remain on how smaller-bodied waterbird species, such as the Terek Sandpiper, manage to consume large crabs, given their relatively narrow, curved bills. While both Whimbrels and Terek Sandpipers possess long bills suitable for extracting crabs from sediments, it might seem implausible for them to swallow a large crab wholly. Biljlsma and de Roder (1991) hypothesized that Terek Sandpipers may only partially consume large crabs by discarding the carapace and feeding solely on the legs. Based on observations, we witnessed Terek Sandpipers and Whimbrels capturing large crabs, striking them against the mudflat to detach the legs, and subsequently discarding the remaining body parts. This feeding behavior aligns with the findings of Piersma (1986), who reported that Whimbrels and Eurasian Curlews remove the legs of Uca sp. larger than 1.0 cm and 1.5 cm, respectively. By selectively feeding on more profitable portions of the crab, these species capitalize on the energetic benefits of larger prey, suggesting that morphological adaptations (e.g., the shape and length of the bill) enable them to handle and process prey items that initially appear too large for their body size.

Herons, egrets and storks possess very long and large bills that allow them to consume fish as their main diet. Their bill structure results in a stronger grip on the prey, especially for fish, since fish can produce a powerful jerk. These species are commonly observed to forage at the edge of receding water during the low tide, where the fish are usually abundant. However, in Tanjung Piai, due to the presence of reclamation land close to the shore, the mudflat area is fully exposed during the peak period of the ebbing tide. As a result, the large waterbird species needed to find alternative prey during those periods, and these species were seen consuming other preys, including crabs, molluscs, and worms. Larger-billed waterbirds presumably have access to a broader range of prey resources, including deeper-buried invertebrates (Zwarts and Wanink 1993, Catry et al. 2014), and ultimately display more efficient generalist behaviour. Therefore, having a long bill might allow these species to exploit a wider range of prey resources and benefit them compared to smaller-billed species with a limited range of preys. This indicates that their morphological differences highly influence the harvestability and accessibility of prey among the coexisting waterbird species.

Large-bodied waterbirds, egrets, and herons preferred to practice pause-travel techniques, while tactile-hunting techniques were preferred by small-bodied waterbirds, including Tibetan Sand-plover, Whimbrel, Terek Sandpiper, Common Sandpiper and Common Redshank. Large waterbirds feed using less active methods compared to smaller waterbirds, which use more dynamic techniques (Richardson et al. 2001). Large-bodied waterbirds with long legs have the ability to feed while standing and walking without sinking into the soft mud. Meanwhile, the smaller waterbirds with shorter legs may permit them to move around more vigorously while probing, including running and turning quickly (Tojo 1996, Marchant et al. 2010). In this study, both egrets and herons were observed hunting close to the water's edge while standing in an upright posture with fully extended necks and waiting for their prey. The same behaviour related to pause-travel techniques was observed by Choi et al. (2017) in Grey Heron. According to McKilligan (2005), the upright posture may benefit the waterbirds in detecting the prey from deeper water than any other behaviour. Feeding close to the water edge might be advantageous, thus resulting in increased penetrability and prey activity (Colwell and Landrum 1993). However, pause-travel species required a longer time spent for feeding than tactile-hunting species. Since pause-travel species can be more vigilant with their heads up and scanning the environment while locating their prey, they spend much of their time being more vigilant instead of foraging (Barbosa 1995, Norazlimi and Ramli 2015). Meanwhile, the tactile-hunting species forage with their heads down and spend much time searching for prey.

According to Davis and Smith (1998) and Nol et al. (2014), the time spent feeding by waterbird is influenced by their body size and dietary differences. Although our results did not show statistically significant differences in feeding time between small-bodied and large-bodied waterbirds, we observed that larger species such as Little Egrets, Grey Herons, Great Egrets, and Lesser Adjutants tends to spend shorter bouts feeding before resting in mangrove trees. One possible explanation is that these larger waterbirds may acquire sufficient energy from fewer, larger prey items, allowing them to forage less frequently and conserve energy during extended rest periods. In contrast, smaller waterbird species, such as the Common Redshank and Tibetan Sand-plover, exhibited more continuous foraging behavior, often for eight to ten minutes at a time. This pattern suggests they rely on smaller, possibly more dispersed prey items, thus necessitating longer active foraging periods. Notably, the actual feeding time of these smaller birds could be underestimated, given that our observations focused on individuals, and each bird may resume foraging multiple times throughout the day.

Additionally, Striated Herons demonstrated a pause-travel foraging technique, scanning prey visually from a stationary position before capturing it. This strategy likely increases their success rate by reducing the chance of startling potential prey, but it also extends the total feeding time required to locate suitable items. These findings align with earlier work (Fauzi and Norazlimi 2021), which observed that Striated Herons walk slowly and occasionally sprint short distances to seize prey, reflecting a trade-off between stealth and active pursuit. Similar behavior was documented in a related species of the same genus, which reportedly stood still for up to 14 minutes before capturing its first prey (Kushlan 2009). Beyond foraging strategy, bill morphology plays a pivotal role in shaping waterbird diets and prey-capture methods. Species with longer, curved bills, such as Whimbrels and Terek Sandpipers, excel at probing into sediment for invertebrates, whereas birds with shorter, robust bills may specialize in visually locating and grasping prey at or near the surface. By linking these morphological traits to the observed foraging behaviors, this study offers a more nuanced understanding of how waterbirds with diverse bill shapes exploit different niches within the same intertidal habitat.

Our observations also suggest that large waterbirds can consume fewer, larger prey items with high profitability, thereby fulfilling their energetic needs in less time. This reduction in foraging effort contrasts with the behavior of smaller waterbirds, which often forage continuously for smaller prey containing lower caloric content. Previous studies (Andrei et al. 2007, Arzel and Elmberg 2015) have similarly shown that small-bodied waterbirds spend more time feeding than large-bodied species. As metabolic rates increase with decreasing body size (Pienkowski and Evans 1984), smaller birds must replenish their energy reserves more frequently (Krijgsveld et al. 2012). Consequently, our findings provide empirical evidence for the link between body size, metabolic demands, and prey size selection in waterbird communities. Collectively, these observations underscore how body size, bill morphology, prey availability, and foraging strategies interact to shape waterbird feeding ecology.

Waterbirds often achieve greater foraging success by feeding in flocks, and two primary hypotheses have been proposed to explain this adaptive behavior. First, group foraging enhances predator avoidance and reduces vigilance costs (Bednekoff and Lima 2005, Burger et al. 2007). Previous work (Quenette 1990, Sansom et al. 2008) suggests that scanning rates for predators decrease as group size increases. Our observations corroborate this concept, particularly in smaller-bodied species such as the Common Redshank, Tibetan Sand-plover, Whimbrel, and Terek Sandpiper. Because these species are more vulnerable to larger predators (e.g., eagles, large-bodied waterbirds), they appear to benefit from a “many eyes” effect (Pulliam 1973) or dilution effect (Hamilton 1971, Kenward 1978) when foraging in flocks, as shared vigilance allows individuals to devote more time to feeding. Secondly, flocking behavior can boost foraging efficiency by directing birds to resource-rich patches (Burger et al. 2007). In this study, we frequently observed intraspecific flocks of Common Redshank and Tibetan Sand-plover returning to the same high-density prey sites, even after temporarily dispersing due to disturbances by larger species such as Milky Storks and Great Egrets. However, flocking may also introduce competitive pressures (Minderman et al. 2006, Beauchamp 2014). When prey availability is limited, the advantages gained through shared vigilance can be offset by increased interference or aggression (Cresswell 1997, Quinn et al. 2006). Indeed, if individuals must spend more time in aggressive interactions or relocating to avoid competition, the net benefit in foraging duration may diminish. Throughout our observations, none of the studied flocks showed a social behaviour of reciprocal altruism where an individual aids another in anticipation that the recipient will return the favor benefitting the individual in future.

Competition for resources generally arises through depletion (consuming the available prey) or interference (Sutherland 1995, Novcic 2018). Our observations indicate that intraspecific flocks of Common Redshank and Tibetan Sand-plover showed higher levels of aggression, including fighting and chasing, likely reflecting their overlap in resource use (Novcic 2018). In contrast, no heterospecific aggression was detected among interspecific flocks, potentially because species like Whimbrels and Terek Sandpipers focus on different prey (e.g., crabs). Moreover, the crab populations in the study sites appeared abundant enough to reduce direct competition between these species. Despite no statistically significant relationship emerging between time spent feeding and feeding success among the observed species, certain patterns suggest a link between foraging strategy, prey size, and success rates. Common Redshank, Common Sandpiper, Tibetan Sand-plover, and Terek Sandpiper, which generally pecked for smaller prey on or near the sediment surface, spent approximately eight to ten minutes in continuous foraging and showed a 60–70% success rate. Their pecking behavior likely benefits from tactile sensory cells in their bills (Piersma et al. 1996, Nebel et al. 2005, du Toit et al. 2020), enabling rapid prey detection (Martins et al. 2013). Nevertheless, because each individual prey item is relatively small and less profitable, these species must make frequent feeding attempts to meet their energy requirements, leading to high feeding and success rates overall.

By contrast, large waterbirds, such as Milky Storks and Egrets, invest less total time in active foraging yet achieve high percentages of successful attempts. Their ability to capture and handle larger prey items stems from their larger bills and overall body size (Durell 2000). These bigger prey items typically offer greater energetic returns (Zwarts and Wanink 1993) but may be inaccessible to smaller birds lacking sufficient bill length or strength. This aligns with the established allometric relationships, in which large birds can reach higher intake rates by effectively exploiting large prey (Zwarts et al. 1990, Nol et al. 2014, Choi et al. 2017). Our findings thereby underscore how morphological advantages enable larger species to gain profitability while expending less total foraging effort. Together, these results highlight the importance of flocking behavior, morphological traits, and varying foraging strategies in shaping waterbird feeding ecology. By examining the interplay between predator avoidance, resource distribution, and interference competition, our study offers insights that extend beyond simple foraging observations, contributing to a more holistic understanding of waterbird community dynamics in intertidal habitats.

## Conclusion

In conclusion, the feeding rate, success rate and percentage of successful attempts of waterbirds in this study are highly influenced by the differences in waterbirds’ morphological traits, feeding techniques engaged and flocking behaviour. All of these are closely related to their time spent on feeding. Large waterbirds recorded a higher percentage of successful attempts, even though lower feeding rates and shorter feeding times. All this evidence pointed out that larger waterbirds are more efficient at feeding and fulfilling their energy requirement. By understanding the feeding efficiency and adaptability of different waterbird species, the conservationists and managers can better anticipate which species are at higher risk and which habitats are most critical to preserve for future.

## Figures and Tables

**Table 1.** Location and protection status of study sites. Pantai Leka (PL), Pantai Mesra Seri Menanti (PSM), Pontian Kechil (PK), Tanjung Piai (TP)

District	Study sites		Coordinates		Protection status
			Latitude	Longitude	
Muar	Pantai Leka	PL 1	1°56'58.35N	102°37'54.94E	Not protected
	Pantai Mesra Seri Menanti	PSM 1	1°53'8.44N	102°40'26.40E	
		PSM 2	1°52'49.43N	102°40'39.41E	
Pontian	Pontian Kechil	PK 1	1°28'35.89N	103°23'22.43E	Not protected
		PK 2	1°28'53.42N	103°23'1.94E	
		PK 3	1°29'14.13N	103°23'8.25E	
	Tanjung Piai	TP 1	1°16'46.40N	103°30'38.99E	Legally protected
		TP 2	1°15'52.76N	103°30'20.31E	
		TP 3	1°16'8.79N	103°30'40.66E	

**Table 2.** List of waterbird species observed. Partially Migrant (PM), Resident (R), Migrant (M), Vagrant (V), Least Concern (LC), Near Threatened (NT), Vulnerable (VU), Endangered (EN).

Family	Species	Common Name	Distribution	IUCN Status (2024)	No. of focal observation
Ardeidae	*Ardeacinerea*	Grey Heron	PM	LC	35
	*Egrettagarzetta*	Little Egret	PM	LC	38
	*Ardeaalba*	Great Egret	PM	LC	30
	*Butoridesstriata*	Striated Heron	PM	LC	35
Charadriidae	*Anarhynchusatrifrons*	Tibetan Sand-plover	M	EN	40
Scolopacidae	*Tringatotanus*	Common Redshank	M	LC	67
	*Numeniusphaeopus*	Whimbrel	M	LC	12
	*Xenuscinereus*	Terek Sandpiper	M	LC	12
	*Actitishypoleucos*	Common Sandpiper	M	LC	13
Ciconiidae	*Leptoptilosjavanicus*	Lesser Adjutant	R	VU	32
	*Mycteriacineria*	Milky Stork	R	EN	14
**Total**					**328**

**Table 3.** Sample size (n), mean and standard error of time spent on feeding and foraging techniques used by waterbirds.

Species	Foraging techniques	Time spent feeding (min)		
		n	Mean	Standard error
Grey Heron	Pause-Travel	26	7.96	1.64
	Tactile Hunting	0	0.00	0.00
	Visual Feeding	9	6.53	2.60
Great Egret	Pause-Travel	26	8.01	1.67
	Tactile Hunting	0	0.00	0.00
	Visual Feeding	4	6.15	3.59
Striated Heron	Pause-Travel	35	11.98	2.24
	Tactile Hunting	0	0.00	0.00
	Visual Feeding	0	0.00	0.00
Little Egret	Pause-Travel	35	7.3	1.30
	Tactile Hunting	3	7.07	5.14
	Visual Feeding	0	0.00	0.00
Lesser Adjutant	Pause-Travel	28	8.72	1.69
	Tactile Hunting	4	6.59	3.82
	Visual Feeding	0	0	0.00
Milky Stork	Pause-Travel	4	11.86	7.03
	Tactile Hunting	5	11.88	6.99
	Visual Feeding	5	12.99	6.81
Tibetan Sand-plover	Pause-Travel	2	6.415	6.51
	Tactile Hunting	38	11.88	6.99
	Visual Feeding	0	0	0.00
Whimbrel	Pause-Travel	0	0	0.00
	Tactile Hunting	12	8.31	2.68
	Visual Feeding	0	0	0.00
Terek Sandpiper	Pause-Travel	0	0	0.00
	Tactile Hunting	12	8.4	2.60
	Visual Feeding	0	0	0.00
Common Sandpiper	Pause-Travel	0	0	0.00
	Tactile Hunting	13	8.03	2.39
	Visual Feeding	0	0	0.00
Common Redshank	Pause-Travel	5	7.09	3.58
	Tactile Hunting	62	9.32	1.32
	Visual Feeding	0	0	0.00

**Table 4.** Values of feeding rate, success rate and percentage of successful attempts between waterbirds.

Species	Feeding Rate (pecks/probes)			Success rate	Percentage of successful attempts (%)
	n	Mean	SE		
Grey Heron	35	5.16	0.751	3.57	89
Great Egret	30	4.32	0.618	3.1	70
Striated Heron	35	1.21	0.324	1.37	90
Little Egret	38	6.32	0.972	3.31	64
Lesser Adjutant	32	5.22	0.859	4.1	89
Milky Stork	14	5.16	0.836	3.3	73
Tibetan Sand-plover	40	12.36	1.641	8.1	67
Whimbrel	12	8.41	1.29	5.53	75
Terek Sandpiper	12	11.08	1.614	8.26	64
Common Sandpiper	13	11.57	1.637	8.0	65
Common Redshank	67	13.65	1.835	9.9	78
